# Evaluation of stiffness feedback for hard nodule identification on a phantom silicone model

**DOI:** 10.1371/journal.pone.0172703

**Published:** 2017-03-01

**Authors:** Min Li, Jelizaveta Konstantinova, Guanghua Xu, Bo He, Vahid Aminzadeh, Jun Xie, Helge Wurdemann, Kaspar Althoefer

**Affiliations:** 1 School of Mechanical Engineering, Xi’an Jiaotong University, Xi’an, Shaanxi, China; 2 State Key Laboratory for Manufacturing Systems Engineering, Xi’an Jiaotong University, Xi’an, Shaanxi, China; 3 School of Engineering and Materials Science, Queen Mary University of London, London, United Kingdom; 4 De Beers Technologies, Maidenhead, United Kingdom; 5 Department of Mechanical Engineering, University College London, London, United Kingdom; 6 Faculty of Science & Engineering, Queen Mary University of London, London, United Kingdom; University of Zaragoza, SPAIN

## Abstract

Haptic information in robotic surgery can significantly improve clinical outcomes and help detect hard soft-tissue inclusions that indicate potential abnormalities. Visual representation of tissue stiffness information is a cost-effective technique. Meanwhile, direct force feedback, although considerably more expensive than visual representation, is an intuitive method of conveying information regarding tissue stiffness to surgeons. In this study, real-time visual stiffness feedback by sliding indentation palpation is proposed, validated, and compared with force feedback involving human subjects. In an experimental tele-manipulation environment, a dynamically updated color map depicting the stiffness of probed soft tissue is presented via a graphical interface. The force feedback is provided, aided by a master haptic device. The haptic device uses data acquired from an F/T sensor attached to the end-effector of a tele-manipulated robot. Hard nodule detection performance is evaluated for 2 modes (force feedback and visual stiffness feedback) of stiffness feedback on an artificial organ containing buried stiff nodules. From this artificial organ, a virtual-environment tissue model is generated based on sliding indentation measurements. Employing this virtual-environment tissue model, we compare the performance of human participants in distinguishing differently sized hard nodules by force feedback and visual stiffness feedback. Results indicate that the proposed distributed visual representation of tissue stiffness can be used effectively for hard nodule identification. The representation can also be used as a sufficient substitute for force feedback in tissue palpation.

## Introduction

Soft-tissue tumors are often localized by conducting intra-operative manual palpation during open surgery. Manual palpation is performed to identify hard nodules by direct tactile sensation. The procedure enables surgeons to gather information regarding the reaction force and help them understand the material properties, particularly soft-tissue stiffness. A stiffer tissue typically indicates the location of tumors [1]. Force and tactile sensing technologies allow the detection of tumors that are invisible on the surface of the soft tissue. These methods also help determine an adequate resection margin. Robot-assisted minimally invasive surgery (RMIS) has been widely studied in recent years [2–6]. However, the lack of haptic feedback presents a significant drawback of current surgical tele-manipulators [1].

In conventional minimally invasive surgery (MIS) in which laparoscopic tools are used, remote palpation can be indirectly conducted using surgical tools (i.e., instrument palpation) [7]. Instrument palpation can also be employed if the RMIS system is equipped with direct force feedback. Note that remote palpation, here, refers to palpation conducted over the distance between the soft tissue and the surgeon introduced by MIS or RMIS. However, slight delays and errors in the system may cause undesired oscillations on the master and slave sides during surgery; such an occurrence may be unsafe for both the surgeon and the patient [8]. Moreover, the trade-off between system transparency—i.e., matching the level of feedback forces and forces applied at the tip of the tool—and system stability is a limitation of direct force feedback [9]. Consequently, feedback methods that do not use direct force feedback have recently drawn significant attention [7,8,10–12]. Miller et al. [7] proposed to display a colored stiffness map on top of the tissue image based on tissue probe; a tactile sensor mounted on the underside of the probe head was used. Yamamoto et al. [13] described a similar approach using a tissue probe with a force sensor. Visual representation of information on tissue stiffness distribution is cost-effective, and force feedback remains an intuitive method of relaying information on tissue stiffness. Visual representation of soft-tissue stiffness distribution has not been compared with force feedback in terms of hard nodule detection performance in a tele-manipulation environment [9,11,14].

As shown in [11,15], if an intra-operative tissue model for palpation based on patient data (by imaging modalities and intra-operative palpation) can be generated using estimated soft-tissue parameters, users can explore the stiffness distribution in a virtual environment by using a haptic device. The technique avoids control issues inherent to systems that provide direct force feedback. Thus, this method represents another approach to intra-operative tumor identification. In our previous study, a virtual tissue model was created based on the (i) surface of an artificial soft tissue reconstructed using a Kinect depth sensor and (ii) organ stiffness distribution achieved during sliding indentation measurements. We preliminary demonstrated the feasibility of this method [16]. With this virtual tissue model, both force feedback and visual stiffness feedback can be provided.

In the current study, we proposed a novel visual stiffness feedback method and evaluated this technique in (1) a tele-manipulation environment and (2) a virtual environment. Palpation by sliding indentation, which can obtain faster the stiffness distribution, replaces the point-to-point uniaxial indentation behavior, as presented in [7,13]. In our proposed system, a graphical interface was employed to display a dynamically updated color map depicting the stiffness of the probed soft tissue. Experiments on hard nodule detection were conducted to validate the usefulness of visual stiffness feedback in a tele-manipulation environment. For the second environment (the virtual environment), a tissue model was generated based on indentation depth/reaction force pairs from indentation measurements that were derived by sliding over a tissue phantom. With this tissue model, we evaluated the performance of human participants in distinguishing the different sizes of hard nodules that are integrated into soft tissue by using our visual stiffness feedback method. This study presents the following contributions:

1. Presentation of color-coded tissue stiffness information based on sliding indentation;
2. Generation of a tissue model that can visually and haptically represent the tissue stiffness distribution of the examined soft tissue;
3. Comparison between the visual representation of soft-tissue stiffness distribution and force feedback for hard nodule detection and hard nodule size discrimination.

This paper is organized as follows: Section 2 elaborates on soft-tissue stiffness feedback for palpation; Section 3 shows the experimental study involving humans; Section 4 presents conclusions.

## Soft-tissue stiffness feedback

### Overview

**Fig 1:**
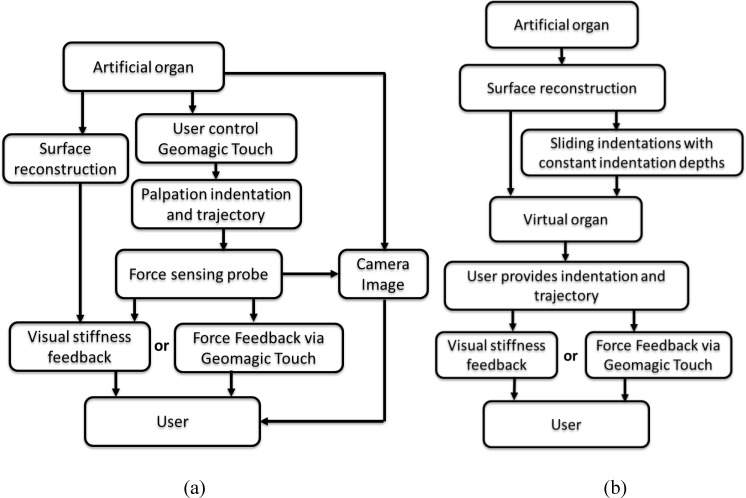
Method flowcharts. (a) Palpation in a tele-manipulation environment; (b) Palpation in a data-driven virtual tissue model. Haptic device Geomagic Touch is used to control the position; Fanuc robot follows the trajectory; force sensing probe scans the silicone phantom; virtual tissue displays on a computer monitor; probe position is displayed in real time; stiffness distribution color map is generated using force and indentation depth data.

**Fig 2:**
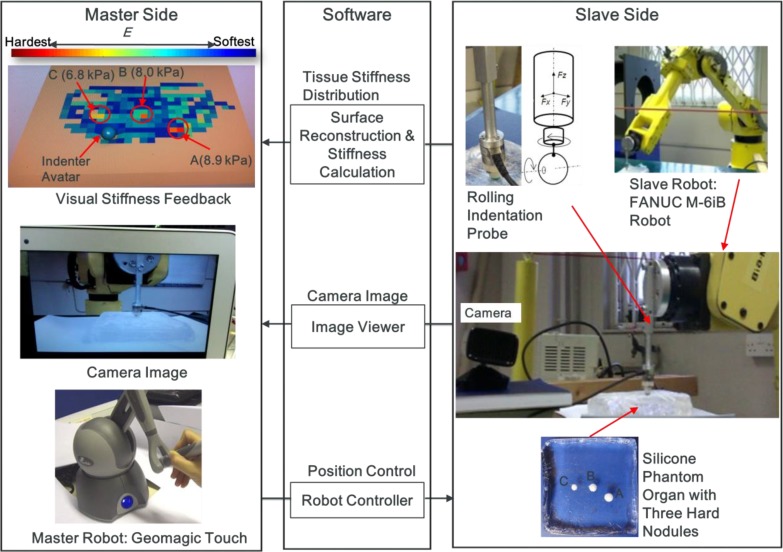
Schematic of the experimental haptic manipulator. The right column shows the slave-side hardware: a camera, a robot arm, a sliding indentation probe, and a silicone phantom tissue. The left column shows the master-side configuration: a live camera image, a visual stiffness display, and force feedback via a haptic device. The middle column lists the software.

A visual stiffness feedback method is proposed and evaluated in both tele-manipulation and virtual environments. Fig 1(A) depicts the procedure flow of tissue palpation in a tele-manipulation environment. Tele-manipulation, here, refers to manipulation overcoming the distance between the soft tissue and the surgeon introduced by RMIS. A master-slave tele-manipulation configuration is created and utilized to simulate the tele-manipulation environment of RMIS. A user applies position control to a robot arm (FANUC robot arm, M-6iB, FANUC Corporation) aided by an input device (Geomagic Touch, 3DS Inc.). A sliding force sensing probe [12,17,18] scans an artificial organ. A virtual model of the artificial organ and the position of the robotic end effector—that is, the tip of the force sensing probe—are displayed in a virtual environment on a computer monitor. A color map representing the stiffness distribution of the artificial organ is generated using the reaction force and indentation depth data. A camera view of the palpation site is also provided on the same computer monitor. In addition, force feedback can be provided via the haptic feedback mechanism of the input device. This experimental platform, designed and created as part of the current study, consisted of the following main components: tele-manipulators (a slave robot and a master robot), a vision system, and a visual stiffness display. Fig 2 depicts the system design of the experimental platform. The software was classified by function into 3 main parts: camera image viewer, reconstruction of virtual soft-tissue surface and stiffness visualization, as well as tele-manipulation. Sensor measurements, including force and position were transmitted from the slave side to enable the virtual soft-tissue surface reconstruction, visual stiffness feedback, and force feedback on the master side. The methodology are presented in detail in subsequent sections.

As shown in Fig 1(B), a virtual soft-tissue model is created based on mechanical characterization of a silicone artificial organ; therefore, our proposed method allows exploring the stiffness distribution via either force feedback or visual stiffness feedback independently of the artificial silicone organ. Sliding indentation [12,17,18] is conducted with controlled indentation depths by using a robot arm to obtain the indentation depth and reaction force pairs of the tissue phantom. During the process, the reaction force data are recorded. A virtual soft-tissue model is then established based on the probing data obtained from the tissue phantom. With the generated tissue model, the user can explore the stiffness distribution apart from the real tissue. Palpation is conducted on this virtual model by force feedback or visual stiffness feedback.

### Tele-manipulator

**Fig 3:**
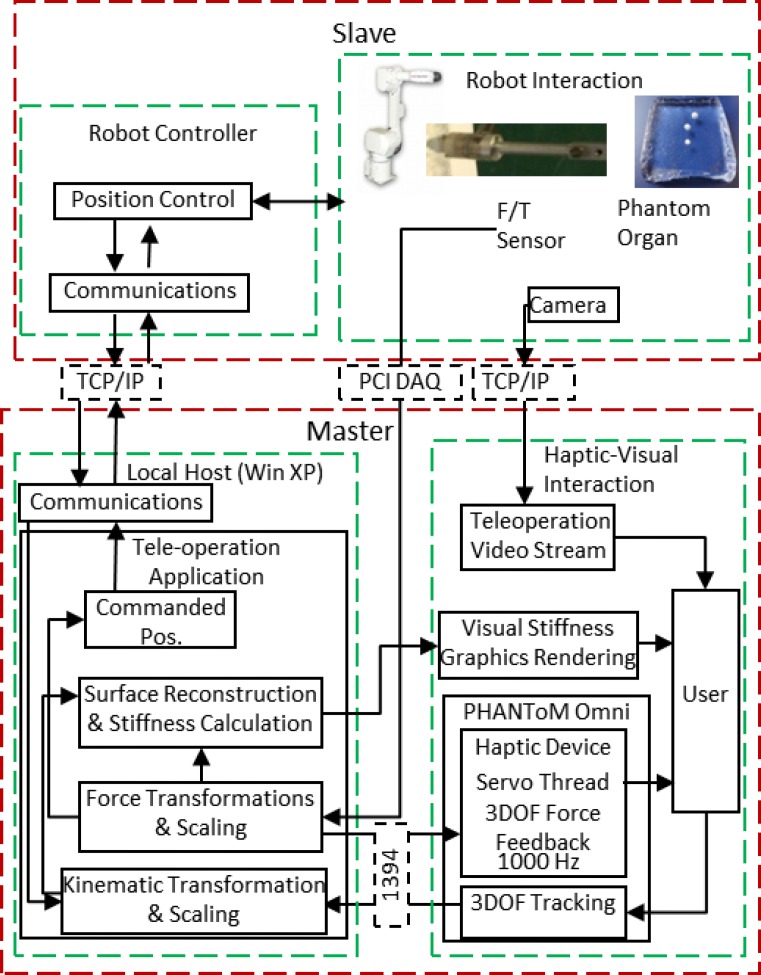
Tele-operation architecture.

A block diagram of the tele-operation architecture is presented in Fig 3. A TCP/IP communication link was used between the master side and the slave side. Both main loops of the master and slave sides were synchronized at a frequency of 21.3 Hz. The position of the master robot end-effector was transmitted to the slave side as an input of the slave robot control loop. Simultaneously, the position information of the slave robot was transmitted to the master side to display the indenter. Haptic and graphic rendering was performed concurrently in separate threads. The haptic device servo thread ran at a frequency of 1000 Hz to provide a satisfactory kinesthetic sense of the physical interaction. Graphic rendering and force data acquisition had the same frequency as that of the main loop of the master side.

Our FANUC M-6iB robot arm was equipped with an R-J3iC controller consisting of an embedded kinematic and dynamic controller optimized for the particular robot. The sequence of the positions provided by the master was passed directly to the trajectory generator of the robot; however, the trajectory generator was set to work in a linear interpolation mode to avoid discontinuity between points.

**Fig 4:**
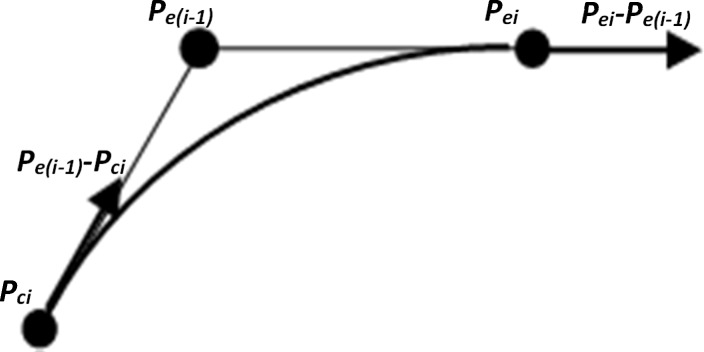
Hermite curve interpolation trajectory generation.

In the linear interpolation mode, the trajectory generated followed the Hermite curve (see Fig 4) with the following set of equations:

$$p(t)=p_{ci}(1-3{\left(t_{i}-t_{c}\right)}^{2}t^{2}+2{\left(t_{i}-t_{c}\right)}^{3}t^{3})+p_{e(i)}(3{\left(t_{i}-t_{c}\right)}^{2}t^{2}-2{\left(t_{i}-t_{c}\right)}^{3}t^{3})+\alpha (p_{e(i-1)}-p_{ci})(t-2{\left(t_{i}-t_{c}\right)}^{2}t^{2}+{\left(t_{i}-t_{c}\right)}^{3}t^{3})+\alpha (p_{ci}-p_{e(i-1)})({\left(t_{i}-t_{c}\right)}^{2}t^{2}+{\left(t_{i}-t_{c}\right)}^{3}t^{3}),$$ (1)

where ***p***_*ei*_ and ***p***_*e(i-1)*_ are 2 consecutive points passed to the trajectory generator, ***p***_*ci*_ is the current location of the robot upon receiving an update on the position ***p***_*ei*_; *α* is a scalar, which determines how strongly the motion of the robot should align with the intermediate point. By adjusting the scalar parameter *α* to a high value, the motion trajectory of the robot from ***p***_*ci*_is parallel to the vector ***p***_*e(i-1)*_*-* ***p***_*ci*_ and ends parallel to the vector ***p***_*ei*_*-* ***p***_*e(i-1)*_. This method allows online and continuous mirroring of the motion of the master–slave manipulator. The choice of parameter *α* is a trade-off between the vibration caused by the robot at higher values and the positioning error at smaller values. In addition, the parameter was chosen empirically based on the trajectories generated; here: *α* = 3.5.

**Fig 5:**
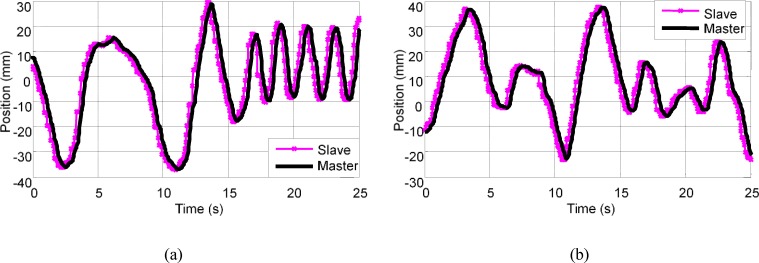
Position response. (a) when no force feedback is applied and (b) when force feedback is applied.

The position response during the random motion was evaluated to determine the performance of the controller. The master and slave position responses were recorded when the master robot was manipulated in a random motion with and without force feedback. Fig 5 shows the experimental results of the recorded master and slave trajectories of the random motion, with and without force feedback. The mean delay of the trajectories without force feedback was 0.25 s with a standard deviation of 0.03 s; meanwhile, the mean delay of the trajectories with force feedback was 0.25 s with a standard deviation of 0.04 s. The slave trajectories matched the master trajectories. No significant difference was indicated between the trajectory tracking with force feedback (mean position error of 0.48 mm) and the trajectory tracking without force feedback (mean position error of 0.53 mm). The encountered delay in position response could have been caused by the data communication between the master and slave robots.

### Force feedback

In the proposed design, force feedback was provided to help the operator form an instinctive impression of the magnitude of tissue stiffness. Instead of using force sensors, bilateral tele-operation controllers and patient-side arm actuator electric currents were used to estimate force [11,14,19]. In [14], estimated forces and measured forces are compared using a force sensor. The result indicates that using estimated forces a tele-operated system can achieve transparency that is sub-optimal relative to what employing a force sensor can achieve. To match the feedback forces presented to the user on the master side with the forces applied on the slave side is particularly challenging for tele-manipulators without force sensors [14]. In the current study, an ATI Nano 17 force sensor (SI-12-0.12, resolution 0.003N with 16-bit data acquisition card) was used to measure force. The separate point (point-to-point) uniaxial indentation method may encounter difficulties for rapid soft-tissue scanning over a large tissue area [12], Consequently, rolling indentation using a force-sensitive wheeled probe for localization were proposed in [12,17,18]. Rolling indentation can rapidly acquire stiffness distribution from trajectories along soft-tissue surfaces. However, this earlier probe with its wheeled indenter needed to be rotated when the rolling direction changes. Thus, an enhanced sliding indentation probe was proposed in [20]. A round-shaped end effector (6 mm in diameter), which was fixed inside the tip of the probe, replaced the indentation wheel of the rolling indentation probe. The tissue surface was lubricated for to reduce the friction while sliding over the tissue surface. A force distribution matrix could be obtained by scanning the soft-tissue surface with the sliding indentation probe. This matrix showed the elastic modulus of the tissue at a given indentation depth with the assumption that the investigated tissue was linear elastic, isotropic, homogeneous, and incompressible [21]. Liu et al. [21] validated the linear elastic assumption in the experimental study. They concluded that this assumption allowed accurate estimate of the elastic modulus when the indentation depth *d*_*in*_ is small (*d*_*in*_< 3.5 mm). Likewise, the indentation speed had no significant impact on the estimated elastic modulus. Therefore, Liu et al. used the equation below to calculate the elastic modulus *E* in their indentation experiments [21]. The same equation was used in the current study.

$$E=\frac{3f(1+\nu )}{8d_{in}\sqrt{rd_{in}}},$$ (2)

where *f* is the interaction force normal to tissue surface, *r* is the radius of the sphere, *d*_*in*_ is the indentation depth and *ν* is the Poisson’s ratio [22]. Soft tissue is assumed to be incompressible. For incompressible material, Poisson’s ratio is 0.5.

For palpation using the tele-manipulation environment, force feedback was applied via a haptic device according to the force sensor measurements on the slave side. For palpation on the data-driven virtual tissue model, the current cursor position and the previous cursor position were first read by the program. In case of a contradiction between the cursor and the original contour of the soft tissue, the indentation depth was calculated. On the basis of the calculated indentation depth, the reaction force was acquired by a look-up table method linearly interpolating between the elements of the table containing pairs of premeasured indentation depth and reaction force from sliding indentation. The maximum executable force in the nominal (orthogonal arms) position of the used 3-DOF of the force feedback device Geomagic Touch was 3.3 N. The force data consisted of 3 components: *f*_*x*_, *f*_*y*_, and *f*_*z*_. The normal reaction force was generated from the value of *f*_*z*_; the horizontal force was the resultant of *f*_*x*_ and *f*_*y*_; and the force direction was calculated based on the difference between the previously updated position *P*_*l*_ and the current position *P*_*c*_.

$$V_{h}\equiv \vec{P_{l}P_{c}},$$ (3)

where *P*_*l*_ is the previous position (*x*_*l*_, *y*_*l*_), *P*_*c*_ is the current position (*x*_*c*_, *y*_*c*_). The horizontal component vector of the force direction was then transformed to a unit vector with the same direction. The tangent force was generated in the direction of the tangent unit vector

$${\hat{V}}_{h}=\frac{V_{h}}{\left|V_{h}\right|},$$ (4)

Where ${\hat{V}}_{h}$ is the unit vector of the force direction. If the force value exceeded the maximum output (3.3 N), the value was retained.

### Visual stiffness feedback

To help an operator acquire a clear view of the stiffness distribution, the surface of a deformable virtual soft tissue with a real-time updated stiffness map was displayed through a graphical interface. A live camera image of the palpation site and a depiction of a separate virtual soft-tissue surface were rendered on a graphical interface in the proposed system. In this study, a phantom tissue with a flat surface was used. The tissue surface height was assumed to be constant. For the uneven tissue surface, a contour scan was needed. This tissue contour scan was discussed in detail in [16].

Deformation of the virtual soft tissue during palpation was displayed in real time by using a geometric deformable soft-tissue model with consideration of the influence of the indenter diameter based on predefined FE modeling; the details of this model were discussed in [23].

**Fig 6:**
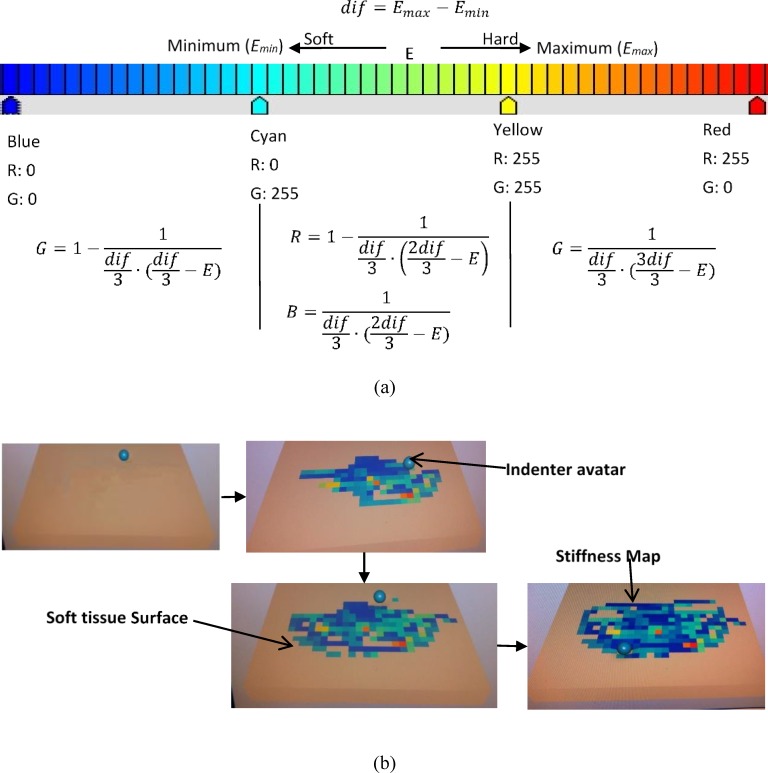
(a) Mapping stiffness data to RGB value and (b) stiffness map generation.

The indentation depth was calculated using the distance between the indenter position (***P***_***0***_) and the nearest triangle planar to the original contour (vertices: ***P***_***1***_, ***P***_***2***_, ***P***_***3***_). The normal vector of the planar ***n*** was obtained from the cross product (***P***_***2***_*-****P***_***1***_) × (***P***_***3***_*-****P***_***1***_). This distance was calculated using the absolute value of the dot product ***v****⋅****n*,** where ***v*** is the vector from ***P***_***0***_ to ***P***_***1***_. With the indentation depth and reaction force, soft-tissue stiffness was derived using Eq (2).The calculated Young’s modulus *E* was stored at its palpated location. This value was then converted to an RGB value by using the minimum and maximum stiffness values in the current storage space (see Fig 6(A)). These pairs of RGB value and palpated location were used to dynamically update the stiffness map. Blue was used to denote the minimum stiffness, whereas red was used to represent the maximum stiffness (see Fig 6(B)).

## Evaluation of the proposed visual stiffness feedback

### Experiment I: Stiffness map generation test in a tele-manipulation environment

**Fig 7:**
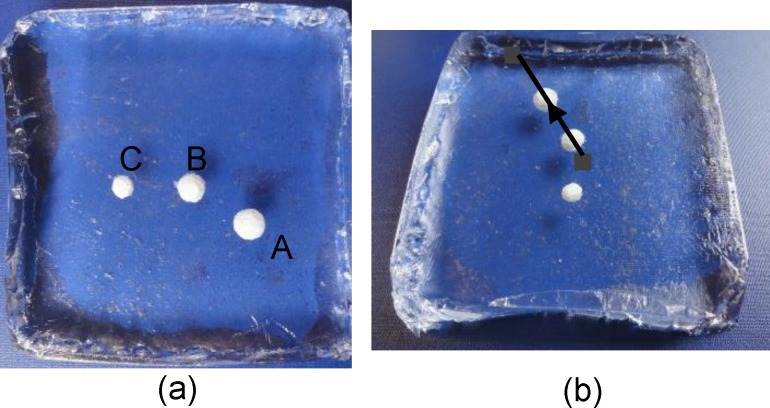
Silicone soft-tissue phantom. (a) the locations of the 3 embedded nodules are highlighted (A, B, C); (b) an operator remotely palpated the phantom tissue by using the same trajectory, which covers nodules A and B, guided by the two black tags.

Prostate tissue was chosen as the organ interest. This experiment aimed to locate the position of a stiff nodule buried under the flat surface of the silicone phantom tissue. The phantom, measuring 120 mm×120 mm×30 mm, contained 3 embedded spherical nodules (A, B, C) (see Fig 7(A)). The silicone block had a flat surface. Identifying T1 stage tumors (up to 20 mm along its largest extension [24]) is highly significant to increase survival rates. In the present study, T1 stage tumors were simulated using artificial tumor models in silicone phantom tissues. Cancerous formations are typically stiffer compared with healthy soft tissues [25]. Therefore, within the scope of the current study, tumors are assumed to be homogeneous and stiffer than the surrounding healthy soft tissues [25]. The phantom was fabricated using RTV6166 (TECHSIL Limited, UK) (A: B = 4: 6, Young’s modulus 7.63 kPa [26]). The nodules were made from STAEDTLER Mars plastic 526 50 (47–50 Shore A, Young’s modulus of about 1.59 MPa). The spherical nodules were 10, 8, and 6 mm in diameter. All nodules were buried at a depth of 6 mm, measured from the top of the nodules to the silicone surface.

**Fig 8:**
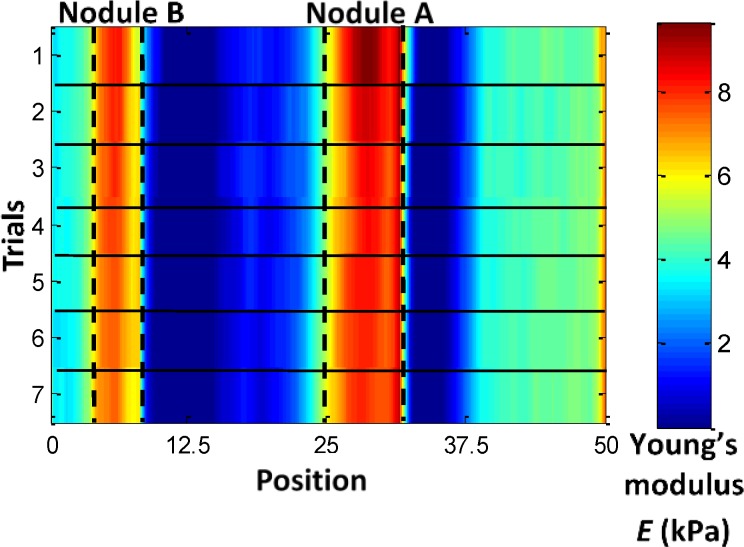
Stiffness map estimated from the perpendicular reaction force along the same trajectory in multiple trials of remote palpation with increased velocity from trials 1 to 7. Nodules A and B are presented in red or orange, whereas other areas are presented in blue or cyan.

To evaluate the robustness of the proposed real-time stiffness calculation and visual feedback generation, the master robot was repeatedly operated in such a way as to palpate the phantom silicone organ along one straight trajectory that covers nodules A and B, using variable velocities. For trials 1 to 7, we used different levels of velocities from the slowest to the fastest achievable speeds. Position and force data were recorded. Fig 7(B) displays the trajectory of motion, which moved across Nodules A and B during this test. Fig 8 shows the stiffness map calculated from the perpendicular reaction force along the same trajectory, in multiple trials of remote palpation, with increasing velocity from trials 1 to 7. Although palpation velocity and indentation depth were not constant in different trials, the stiffness maps produced were similar. The Young’s moduli of the artificial organ are comparable to prostate tissue elasticity properties shown in [27]. This result proves the robustness of the presented method in calculating real-time stiffness. Liu et al. [21] found that the indentation speed exhibited no significant impact on the estimated elastic modulus in their rolling indentation experiments. In the current study, the stiffness map generation test proved that the calculated stiffness is similar for different palpation velocities, verifying the findings by Liu et al. [21]. The accuracy of the contour of soft tissue could affect the estimation of the indentation depth and further influence the accuracy of the stiffness. Thus, a tissue silicone phantom with a flat surface was used in the present study. For uneven tissue surfaces, as shown in our previous study [16], we propose to provide 3D reconstruction of the soft tissue using a Kinect sensor.

### Experiment II: User study on nodule detection

One left-handed and 9 right-handed subjects aged 23 to 42 years were asked to perform a palpation task with the tele-operation system by the 2 feedback modes described earlier. Since the proposed method could possibly affect the way palpation is performed and experienced surgeons typically have special techniques for palpation, we wanted to use subjects that were not familiar with palpation. Three of the subjects had previous experience with the system, and the remaining subjects had little or no experience with haptic feedback devices. None had palpation experience or a medical background. Information on the participants is presented in Table 1. We would like to conduct experiments with more participants with different levels of palpation expertise in our future study. This work was approved by the King’s College London Biomedical Sciences, Dentistry, Medicine and Natural & Mathematical Sciences Research Ethics Subcommittee (BDM/10/11-95). Participants signed a written consent form before the experiment.

**Table 1:** Overview of participant demographics and experience in the palpation experiment with the tele-operation system (Experiment II).

	Detail
**Age range**	23–42
**Average age**	27.8
**Gender**	♀: 3; ♂: 7
**Handedness**	R: 9; L: 1
**Tele-manipulator**	3
**Palpation experience**	0

All trials were performed by participants controlling the slave robot to palpate the silicone phantom tissue with the use of the Geomagic Touch stylus. To prevent damage to the phantom tissue during palpation and maintain the palpation force within a safe range, a limit on the indentation depth (6 mm) was set. The participants viewed the environment on a graphical interface via a computer monitor. The surface of the phantom tissue was covered by a transparent plastic film and lubricated to reduce friction and dragging forces (Boots Pharmaceuticals Intrasound Gel). The surface of the phantom was palpated continuously to allow fast scanning and stiffness representation. The palpation trials were continuously conducted using the sliding indentation probe.

The first participant was asked to choose either of the 2 feedback modes (force feedback and visual stiffness feedback) to start the experiment. The second participant started with the other feedback mode. The visual stiffness feedback consisted of a representation of a deformable virtual soft-tissue surface. This tissue surface was overlaid with a dynamically updated color stiffness map. Prior to the first trial, the participants were allowed a trial on a different phantom tissue. Force data and time elapsed during each trial were recorded for each participant. The orientation of the phantom tissue was changed for each trial.

**Fig 9:**
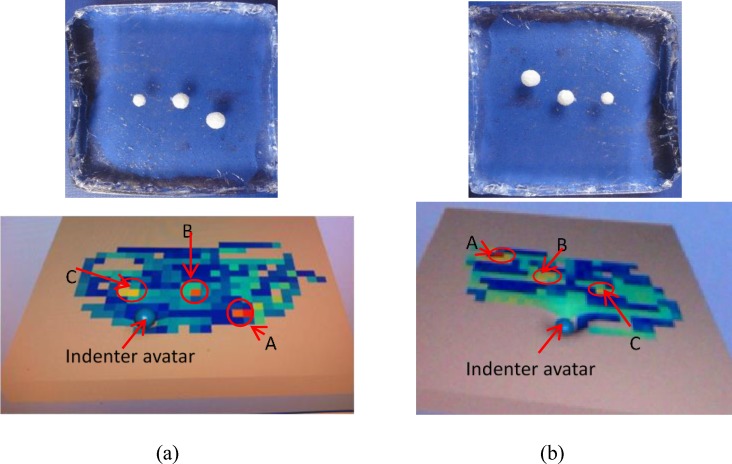
Two stiffness maps generated during 2 trials by visual stiffness feedback. The upper maps show the orientations of the artificial organ, whereas the lower maps show the corresponding stiffness.

Fig 9 shows 2 stiffness maps obtained during a palpation trial by visual stiffness feedback. As indicated by the color stiffness maps, the 3 nodules were detected at their accurate locations. The largest nodule, nodule A, is marked in red color; the middle-sized nodule, nodule B, in orange; and the smallest nodule, nodule C, in yellow.

**Fig 10:**
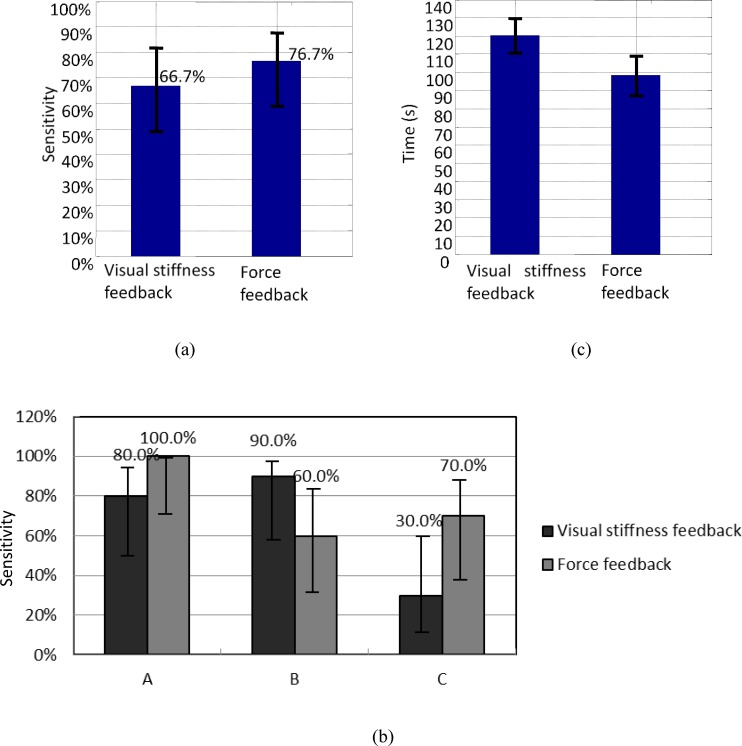
Nodule detection results. (a) Nodule detection sensitivities of visual stiffness feedback and force feedback and Wilson score intervals at a 95% confidence level; (b) Detection sensitivities of each nodule and Wilson score intervals at a 95% confidence level; (c) Time elapsed to determine nodule locations by visual stiffness feedback and force feedback in a tele-manipulation environment: data were averaged over all 10 subjects, and standard error bars are shown (The standard error of the mean is the standard deviation of the sampling distribution of a statistic [34]; it is an indicator of result precision).

The magnitude of the palpation force recorded by the system ranged from 0 to 3.24 N. The average palpation force of the participants was 2.27 N. Sensitivity *Se* [28], which relates to the ability of the test to identify positive results, was defined as the sum over all the *n* trials of the true positives *TP* divided by the sum of false negatives *FN* and *TP*. Fig 10(A) presents the nodule detection sensitivities obtained using the different feedback methods. Wilson score intervals [29], which exhibit good properties even for a small number of trials (less than 30), were calculated at a 95% confidence level. In the current study, since the confidence level of 95%, the error *α* was 5%. The sample size was 30 (3 nodules × 10 participants). The nodule detection *Se* values were as follows: 66.7% (95% confidence interval: 48.8%–80.8%) for visual feedback, 76.7% (95% confidence interval: 59.1%–88.2%) for force feedback, and 73.3% (95% confidence interval: 55.5%–85.8%) visual + force feedback. Fig 10(B) presents the nodule detection sensitivities of nodules A, B, and C. The middle-sized nodule B seems more easily detected by visual feedback than force feedback (visual vs. force: 90.0% vs. 60.0%), whereas the largest and smallest nodules (visual/force: 80.0% vs. 100%, 30.0% vs. 70.0%) were more easily detected by force feedback than visual feedback. The significance of the difference in sensitivity *Se* between paired tests was examined by comparing the observed probabilities (*p*_*1*_ and *p*_*2*_) with a combined interval *CI* [30]. If |*p*_*1*_ *–p*_*2*_| >*CI*, then a significant difference between the two tests was present. However, in the present study, CI was 0.225 and |*p*_*1*_ *–p*_*2*_| was 0.100; given that |*p*_*1*_ *–p*_*2*_| <*CI*, we conclude that no significant difference in nodule detection *Se* was indicated between these two methods.

Fig 10(C) shows the time elapsed for nodule detection. In general, the proposed tele-manipulator was time-efficient for tumor identification, with 107.6 s as the average time for all trials (As shown in [23], participants needed 106.2 s to palpate a similar artificial silicone organ).Wilcoxon matched-pairs signed-rank test [31,32]was used to compare the time elapsed by each pair of feedback method modes. Using this test, one can determine whether the sample size distributions are identical without checking the normal distribution [33]. The significance level of 0.05 was checked. In the present study, *n*_*r*_ = 10, *W =* 12, and *W*_*critical*_ *=* 8. Given *W*>*W*_*critical*.,_ no significant difference in the time required to complete nodule detection was indicated between the tests.

The proposed haptic tele-manipulator provides more flexibility for tissue stiffness feedback modes. The experiments showed no significant difference in nodule detection rates among the methods used. Thus, in cases when direct force feedback could not be achieved, visual stiffness feedback can be used to provide tissue property information to surgeons. An example of these cases is when haptic feedback devices cannot be integrated in the surgical tele-operator.

The experimental results in [14] show that surgeons with extended da Vinci experience performed better when force feedback was used in tele-operated palpation. This finding support previous findings, which show that the benefits of force feedback depends on the RMIS experience of the surgeon [35,36]. Most participants of the current study had no experience with haptic feedback or tele-manipulators, suggesting that performance can be further improved with more practice.

### Experiment III: User study on nodule size discrimination

**Fig 11:**
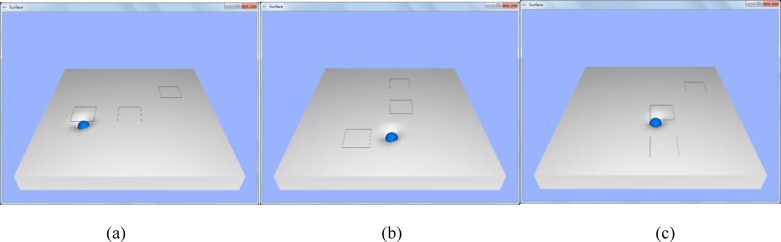
User interfaces of the nodule size discrimination experiment. The buried nodule is marked using black hollow squares; the orientation of the phantom tissue is changed for each trial.

We also evaluated the performance of human participants in distinguishing different sizes of hard nodules that were integrated into the soft tissue to further investigate the effectiveness of the stiffness feedback. The palpation experiments were conducted on a data-driven virtual tissue model that was created based on indentation depth and reaction force pairs of the artificial silicone organ. For each trial, the buried nodules (A>B>C) were marked on the surface using black hollow squares (see Fig 11). A total of 22 participants were asked to perform a palpation task. Details on the participants are presented in Table 2. This study was approved by the institutional review board of Xi’an Jiaotong University. Participants signed a written consent form before the experiment. During the user study, participants were asked to compare the stiffness of the 3 locations and rank the stiffness levels. The order of the stiffness levels of the 3 hard nodules and the elapsed time were then recorded. The first participant was asked to choose either force feedback or visual stiffness feedback to start the experiment, the second participant started with the other feedback mode, and so on. The orientation of the phantom tissue was changed for each trial.

**Table 2:** Overview of participant demographics and experience in the virtual palpation experiment (Experiment III).

	Detail
**Age range**	21–25
**Average age**	22.8
**Gender**	♀: 2; ♂: 20
**Handedness**	R: 20; L: 2
**Tele-manipulator**	0
**Palpation experience**	0

**Fig 12:**
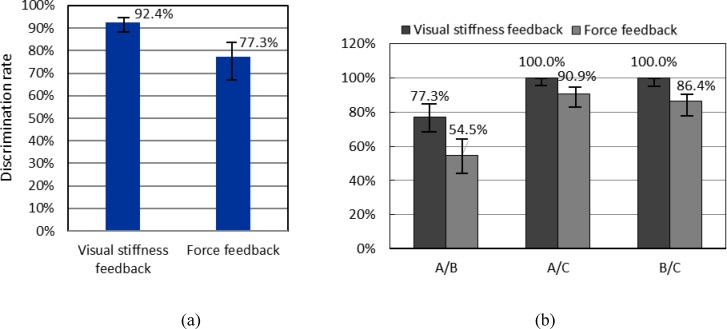
Results of nodule size discrimination. (a) Overall discrimination rates of nodule sizes by the two stiffness feedback modes and their Wilson score intervals at a 95% confidence level; (b) Discrimination rates of the nodule sizes of each nodule pair and their Wilson score intervals at a 95% confidence level.

As shown in Fig 12, when visual stiffness feedback was applied, 77.3% (95% confidence interval: 67.5%–84.7%) of the participants could correctly discriminate nodules A and B, and 100% (95% confidence interval: 95.9%–100%) could correctly discriminate nodules A and C as well as B and C. For force feedback, the ratios were 54.5% (95% confidence interval: 44.2%–64.5%) for nodules A and B, 90.9% (95% confidence interval: 83.1%–95.3%) for nodules A and C, and 86.4% (95% confidence interval: 77.7%–90.0%) for B and C. The overall size discrimination rates for visual stiffness and force feedback were 92.4% (95% confidence interval: 88.6%–95.0%) and 77.3% (95% confidence interval: 71.9%–81.9%), respectively. No pairs of confidence intervals overlapped. Therefore, a significant difference was indicated between each pair of size discrimination rates for the 2 feedback methods. By visual stiffness feedback, the average perceived size order of nodules A, B, and C were 1.14, 1.86, and 3, respectively, which matched well with the actual order of the nodule sizes. For force feedback, the figures were 1.55, 1.68, and 2.77.

## Conclusion

This study evaluated the visual representation of soft-tissue stiffness distribution in identifying tumors by comparing with the force feedback method in both tele-manipulation and virtual environments. Visual stiffness feedback was provided by refreshing the color of a reconstructed soft-tissue surface representation on a graphical interface. Soft-tissue stiffness data estimated in real time were used. Two stiffness feedback modes for soft-tissue palpation were investigated: (1) force feedback and (2) visual stiffness feedback. In a tele-manipulation environment, force feedback was provided by a haptic device using the measurements from an F/T sensor attached to a sliding indentation probe with which the user could probe the surface continuously. A total of 10 participants used the tele-manipulator to palpate an artificial organ with hard nodules embedded. We also evaluated the performance of 22 human participants in distinguishing hard nodules of different sizes. We used a virtual tissue model that was based on a mechanical characterization of the artificial organ. Results indicated that subjects could localize nodules by both feedback modes; no significant difference in nodule detection sensitivity and time elapsed for hard nodule detection was found between these two feedback modes; compared with force feedback, visual stiffness feedback showed a significantly higher nodule size discrimination rates. Stiffness feedback was confirmed to be an effective method. To conclude, our proposed visual stiffness feedback method can be used for tissue palpation if the force feedback method is not available.

## Supporting information

S1 Table Nodule detection results. (DOCX)

S2 Table Nodule size orders recognized by participants. (DOCX)
