# Supplementary material for: Evaluation of stiffness feedback for hard nodule identification on a phantom silicone model
Source: PLoS One. 2017 Mar 1;12(3):e0172703. doi: 10.1371/journal.pone.0172703 (PMC5383005; doi:10.1371/journal.pone.0172703)
Supplement: S1 Table — (DOCX) [file pone.0172703.s001.docx]

**S1 Table. Nodule detection results**

|  | **Visual stiffness feedback** | | | | **Force feedback** | | | |
| --- | --- | --- | --- | --- | --- | --- | --- | --- |
| **Participant** | A | B | C | Time (s) | A | B | C | Time (s) |
| **1** | 1 | 1 | 1 | 87 | 1 | 1 | 1 | 58 |
| **2** | 1 | 1 | 0 | 77 | 1 | 0 | 1 | 90 |
| **3** | 1 | 1 | 1 | 120 | 1 | 0 | 1 | 130 |
| **4** | 1 | 1 | 0 | 150 | 1 | 1 | 1 | 91 |
| **5** | 0 | 1 | 0 | 112 | 1 | 1 | 1 | 40 |
| **6** | 1 | 1 | 0 | 91 | 1 | 1 | 0 | 51 |
| **7** | 0 | 1 | 1 | 115 | 1 | 1 | 0 | 110 |
| **8** | 1 | 1 | 0 | 156 | 1 | 0 | 1 | 150 |
| **9** | 1 | 1 | 0 | 116 | 1 | 0 | 0 | 124 |
| **10** | 1 | 0 | 0 | 180 | 1 | 1 | 1 | 139 |
