# Supplementary material for: Evaluation of stiffness feedback for hard nodule identification on a phantom silicone model
Source: PLoS One. 2017 Mar 1;12(3):e0172703. doi: 10.1371/journal.pone.0172703 (PMC5383005; doi:10.1371/journal.pone.0172703)
Supplement: S2 Table — (DOCX) [file pone.0172703.s002.docx]

**S2 Table.** **Nodule size orders recognized by participants**

|  | **Visual stiffness feedback** | | | **Force feedback** | | |
| --- | --- | --- | --- | --- | --- | --- |
| **Participant** | A | B | C | A | B | C |
| **1** | 1 | 2 | 3 | 1 | 3 | 2 |
| **2** | 1 | 2 | 3 | 1 | 2 | 3 |
| **3** | 1 | 2 | 3 | 2 | 1 | 3 |
| **4** | 1 | 2 | 3 | 2 | 3 | 1 |
| **5** | 1 | 2 | 3 | 1 | 2 | 3 |
| **6** | 1 | 2 | 3 | 2 | 1 | 3 |
| **7** | 1 | 2 | 3 | 2 | 1 | 3 |
| **8** | 1 | 2 | 3 | 1 | 2 | 3 |
| **9** | 1 | 2 | 3 | 1 | 2 | 3 |
| **10** | 2 | 1 | 3 | 2 | 1 | 3 |
| **11** | 1 | 2 | 3 | 2 | 1 | 3 |
| **12** | 1 | 2 | 3 | 2 | 3 | 1 |
| **13** | 2 | 1 | 3 | 1 | 2 | 3 |
| **14** | 1 | 2 | 3 | 1 | 2 | 3 |
| **15** | 1 | 2 | 3 | 1 | 2 | 3 |
| **16** | 1 | 2 | 3 | 2 | 1 | 3 |
| **17** | 1 | 2 | 3 | 2 | 1 | 3 |
| **18** | 1 | 2 | 3 | 2 | 1 | 3 |
| **19** | 1 | 2 | 3 | 1 | 2 | 3 |
| **20** | 2 | 1 | 3 | 1 | 2 | 3 |
| **21** | 1 | 2 | 3 | 2 | 1 | 3 |
| **22** | 1 | 2 | 3 | 2 | 1 | 3 |
